# Sodium Chloride Inhibits the Growth and Infective Capacity of the Amphibian Chytrid Fungus and Increases Host Survival Rates

**DOI:** 10.1371/journal.pone.0036942

**Published:** 2012-05-10

**Authors:** Michelle Pirrie Stockwell, John Clulow, Michael Joseph Mahony

**Affiliations:** School of Environmental and Life Sciences, The University of Newcastle, Callaghan, New South Wales, Australia; Louisiana State University, United States of America

## Abstract

The amphibian chytrid fungus *Batrachochytrium dendrobatidis* is a recently emerged pathogen that causes the infectious disease chytridiomycosis and has been implicated as a contributing factor in the global amphibian decline. Since its discovery, research has been focused on developing various methods of mitigating the impact of chytridiomycosis on amphibian hosts but little attention has been given to the role of antifungal agents that could be added to the host's environment. Sodium chloride is a known antifungal agent used routinely in the aquaculture industry and this study investigates its potential for use as a disease management tool in amphibian conservation. The effect of 0–5 ppt NaCl on the growth, motility and survival of the chytrid fungus when grown in culture media and its effect on the growth, infection load and survivorship of infected Peron's tree frogs (*Litoria peronii*) in captivity, was investigated. The results reveal that these concentrations do not negatively affect the survival of the host or the pathogen. However, concentrations greater than 3 ppt significantly reduced the growth and motility of the chytrid fungus compared to 0 ppt. Concentrations of 1–4 ppt NaCl were also associated with significantly lower host infection loads while infected hosts exposed to 3 and 4 ppt NaCl were found to have significantly higher survival rates. These results support the potential for NaCl to be used as an environmentally distributed antifungal agent for the prevention of chytridiomycosis in susceptible amphibian hosts. However, further research is required to identify any negative effects of salt exposure on both target and non-target organisms prior to implementation.

## Introduction

The chytrid fungus *Batrachochytrium dendrobatidis* is a recently emerged pathogen that causes the fatal disease chytridiomycosis in amphibian hosts and has been implicated as a causal agent in the decline and extinction of over 200 amphibian species [1], [2], [3]. The chytrid fungus has two distinct life stages, the motile zoospore stage that disperses through water bodies to locate a host and then encysts to become the growing zoosporangia stage [4]. It infects the keratinized epidermal tissue of post-metamorphic amphibians which disrupts osmoregulatory function, causing electrolyte imbalances and cardiac arrest [5], [6]. It also infects the keratinised mouthparts of tadpoles which can impede feeding ability and reduce development rates [7], but does not result in disease [2]. This pathogen is now well established in amphibian communities around the world [8] and although a free-living life stage has not been found, it can persist in reservoir populations of tadpoles and less susceptible species [9], [10], [11], [12], [13]. The widespread distribution of this pathogen and the presence of reservoir hosts mean that susceptible populations remain vulnerable to disease outbreaks. As a result, reversing amphibian population declines will not be possible until a way of managing the effect of chytridiomycosis is found.

Since the discovery of the amphibian chytrid fungus, research has been directed toward the identification of strategies to mitigate its impact, focusing on the manipulation of host immune responses [14], [15], [16], [17], [18], [19], immunisation [20], [21], antifungal treatments [22], [23], [24] and climatic refuges [25], [26], [27]. However, few studies have investigated the role of antifungal agents that could be added to the host's environment. Sodium chloride (NaCl) salt is a known antifungal agent that acts by altering osmotic gradients, forcing organisms to expend energy in osmoregulation, diverting it away from growth [28]. At concentrations beyond tolerance limits, the osmoregulatory processes are overloaded and death occurs. Exposure to dissolved NaCl is an accepted treatment for numerous types of fungal infections in amphibians [29] and is used extensively as an antifungal prophylactic within the aquaculture industry [30].

The addition of 1–5 ppt NaCl to water bodies is known to lower the prevalence of fungal infections in freshwater fish species, increasing both hatching and survival rates [30], [31], [32], [33]. However, in nearly all cases, higher NaCl concentrations have negative effects on the fish themselves. Therefore, in order for NaCl to be an effective antifungal agent, the host must have higher tolerance limits than the pathogen. The exposure of *B. dendrobatidis* to sodium chloride results in a loss of motility and stunted growth at concentrations of 6.25 ppt NaCl and death has been observed at 12.5 [34] and 50 ppt [35]. However, the effect of lower concentrations has not been investigated. The tolerance limits of amphibians to NaCl are generally low but vary with life history and local environmental adaptations [36].

This study aims to investigate the inhibitory properties of 1–5 ppt NaCl exposure on the pathogen, the host and the outcome of infection. The effect on the chytrid fungus was investigated by determining the relative abundance of motile zoospores, developing zoosporangia and mature zoosporangia, as well as the viability of non-motile zoospores, in culture after 11 days exposure to NaCl. The effect on susceptible frog hosts was investigated by comparing growth, infection load and survivorship following exposure to NaCl for 2 months. The results reveal that NaCl exposure has an inhibitory effect on zoosporangia growth, zoospore motility, infection load and host mortality rate.

## Methods

### Ethics Statement

All work was conducted in accordance with the Australian Government National Health and Medical Research Councils Code of Practice for the Care and Use of Animals for Scientific Purposes and under the approval of the University of Newcastle's Animal Care and Ethics Committee, project number 989 1106.

### Culture Experiment

To test the effect of salt on chytrid growth and survival, TGhL liquid media was made (16 g tryptone, 2 g gelatine hydrolysate, 4 g peptonised milk, 1 L deionised water) with the addition of naturally evaporated sea salt (99.6% NaCl) to equal 0, 1, 2, 3, 4 or 5 ppt. 900 µl of each was added to 48-well flat-bottom culture plates, with each concentration replicated 10 times. 100 µl (∼1.5×10^6^ zoospores) of actively growing chytrid culture (Gibbo River-Llesueuri-00-LB1) was then also added to each well and incubated at 22°C for 11 days. The amount of growth on the bottom of each well was then measured within an ocular frame (76×42 µm) at 400× magnification under an inverted microscope (Axiovert 35, Zeiss). The number of developing and mature zoosporangia within the frame were counted from three random positions in each well and averaged.

Developing zoosporangia were classified as any stage between a germling, showing visible signs of growth and rhizoid formation, to an enlarged cell with contents defined into individual zoospores but lacking a visibly open discharge tube [4]. Mature zoosporangia were fully developed with either a visibly open discharge tube or at least some zoospores having exited the cell. Empty zoosporangia were also included in this category. Finally, the number of non-motile and motile zoospores in each well was counted as the number in the ocular frame or the number passing through the ocular frame within 10 seconds, repeated three times within each well and averaged. A relative measure of the proportion of zoospores that were motile was then calculated for each well as the number moving through the frame divided by the sum of the number counted in, and moving through, the frame. Comparison of mean counts between the NaCl treatment groups were conducted using analysis of variance (ANOVA) followed by *post-hoc* Tukey tests.

To quantify the survival of zoospores in each treatment group, 950 µl of media was removed from each well and the remaining cells stained with a SYBR14/propidium iodide viability stain following a protocol specifically optimised for the chytrid fungus [37]. The number of non-motile live and dead zoospores in the ocular frame was then counted based on colour differentiation. The average of three counts per well was determined and the proportion of dead cells calculated and compared between treatment groups using ANOVA.

### Infection Experiment

To investigate the effect of salt and exposure to the chytrid fungus on the body size, infection load and survival of susceptible hosts, a 2×6 factorial experiment was conducted using juvenile Peron's tree frogs *Litoria peronii*, a species known to be susceptible to infection [38]. Individuals were placed into 15 L plastic aquaria with gravel substrates sloping into 2 L of aged tap water containing either 0, 1, 2, 3, 4 or 5 ppt sea salt. 1 mL (∼1.0×10^7^ zoospores) of actively growing chytrid culture was then added to half of each salt treatment tanks and each treatment group had 11 replicates. Tanks were positioned randomly within a constant temperature room and maintained at 22°C under a 12 hour light regime. Water levels were topped up daily where required to maintain salt concentrations and half water changes were conducted fortnightly. Each frog was provided with three small crickets three times a week.

Prior to the start of the experiment, the body mass of each individual was measured and its righting reflex tested by placing the animal on its back and recording the time taken to right itself four times. For ethical reasons, animals were required to be euthanized if they reached the terminal stages of chytridiomycosis which can be identified by lethargy and a lack of responsiveness associated with slow or non-existent righting reflex [39]. Therefore, following inoculation of tanks with the chytrid fungus, each animal was monitored daily. Where individuals appeared lethargic and/or did not respond to external stimuli, their righting reflex was again tested and compared with the initial base-line values. If the time taken to right itself was more than twice that of the baseline value it was again tested 2 and 4 hours later. If no improvement was noted the animal was then euthanized by immersion in a 0.4% tricaine methanesulphonate (MS-222) solution. For the purposes of analysis, these animals were considered to have died from chytridiomycosis. Animals were monitored for 120 days, at which point the experiment was terminated. The effect of salt on survival rate was analysed using log-rank mantel Cox tests in Kaplan-Meier survival analysis.

Two months following inoculation with the chytrid fungus, the body mass of each animal was measured and the amount of weight gained from the start of the experiment determined. An epidermal swab was also taken by wiping the swab over the animal's ventral surface in a standardised manner. The quantification of chytrid fungus on each swab was then done following standard protocols for a qPCR TaqMan assay [40] using a Rotor Gene 6000 real time DNA amplification system (Corbett Life Science). Each swab was analysed in triplicate and included an internal positive control to test for false negatives. Where all three replicates for each survey gave a positive result for the presence of the chytrid fungus and there was no indication of inhibition, the number of genomic equivalents (GE) detected at a standardised cycle threshold was summarised as the geometric mean for each swab. This value provides a relative measure of the infection load in each individual. The effect of salt on infection load in animals exposed to the chytrid fungus was determined using a Kruskal-Wallis H test and Mann-Whitney U tests were then used *post-hoc* to determine which groups were significantly different. Finally, the effect of salt and chytrid exposure on the amount of weight gained over the first two months of the experiment was determined using a univariate general linear model.

## Results

### Culture Experiment

**Figure 1:**
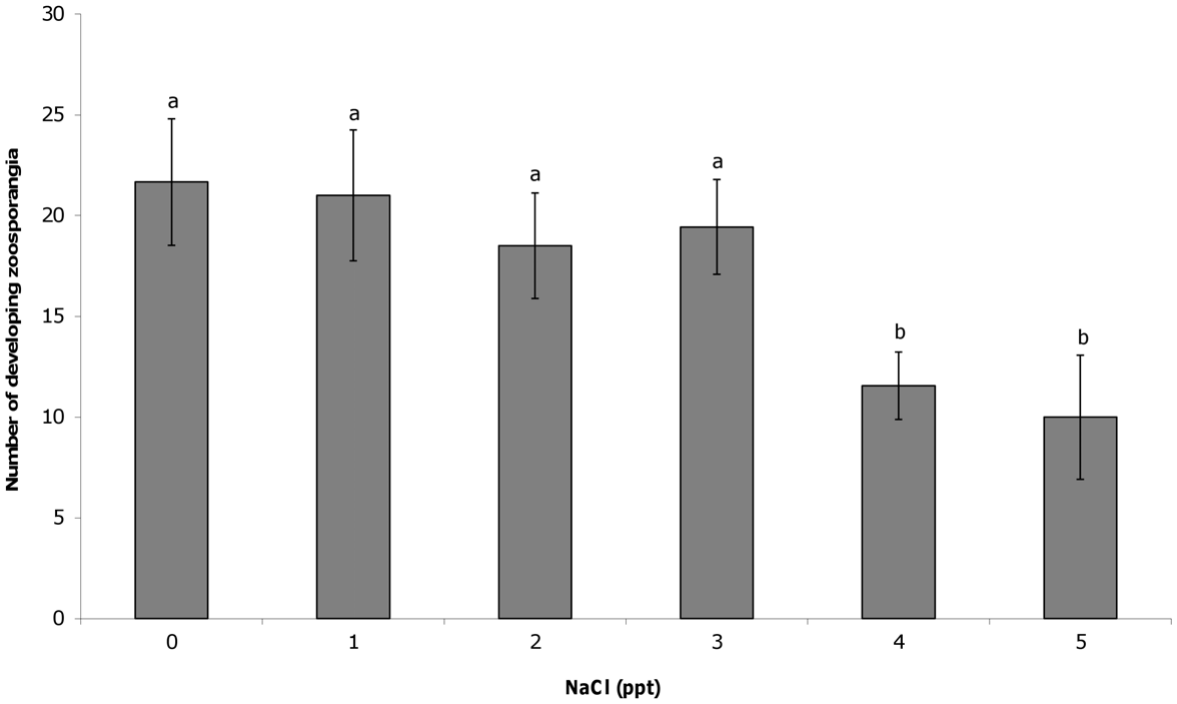
Effect of NaCl on developing zoosporangia. The mean (±SE) number of developing *Batrachochytrium dendrobatidis* zoosporangia counted within an ocular frame at 400× magnification when grown for 11 days in liquid media with varying salt concentrations. Different letters above bars indicate significant differences were found between groups.

**Figure 2:**
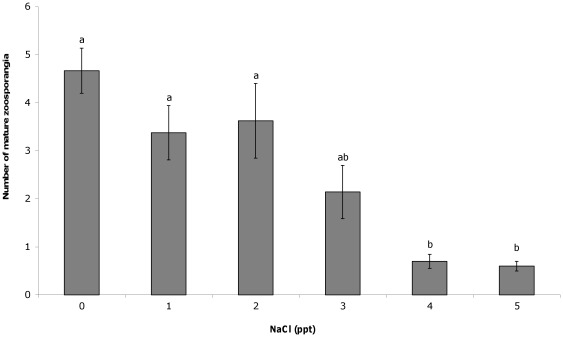
Effect of NaCl on mature zoosporangia. The mean (±SE) number of mature *Batrachochytrium dendrobatidis* zoosporangia counted within an ocular frame at 400× magnification when grown for 11 days in liquid media with varying salt concentrations. Different letters above bars indicate significant differences were found between groups.

**Figure 3:**
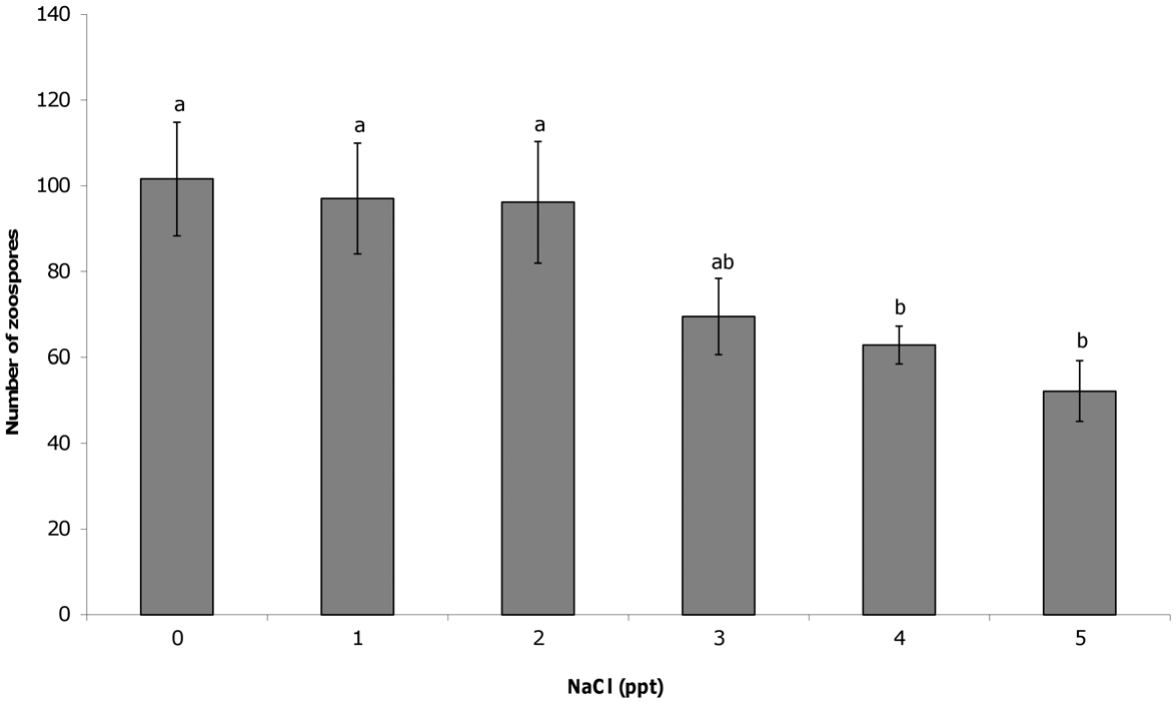
Effect of NaCl on chytrid zoospores. The mean (±SE) number of *Batrachochytrium dendrobatidis* zoospores that were counted in or moving through an ocular frame at 400× magnification after 11 days growth in liquid media with varying salt concentrations. Different letters above bars indicate significant differences were found between groups.

**Figure 4:**
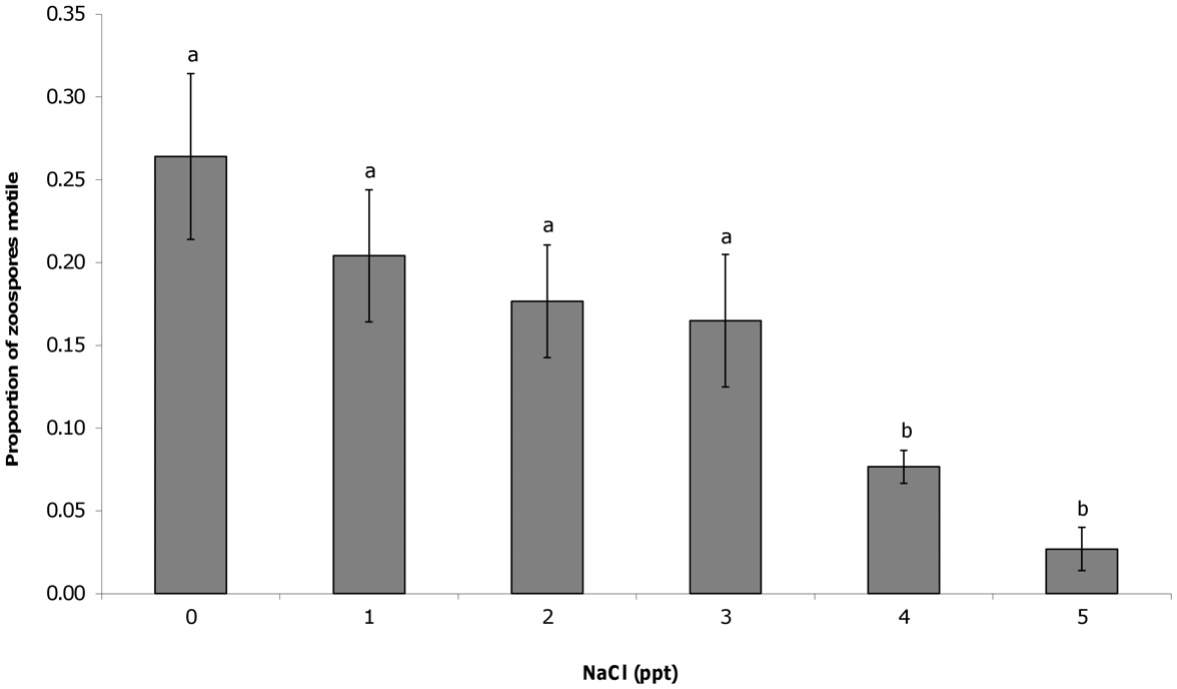
Effect of NaCl on zoospore motility. The mean (±SE) proportion of *Batrachochytrium dendrobatidis* zoospores that were motile when counted through an ocular frame at 400× magnification after 11 days growth in liquid media with varying salt concentrations. Different letters above bars indicate significant differences were found between groups.

The addition of salt to liquid culture media resulted in significant differences in the number of cells counted 11 days after inoculation. At NaCl concentrations greater than 3 ppt, significantly fewer developing zoosporangia (F(5,44) = 4.05, P = 0.004; Figure 1) mature zoosporangia (F(5,44) = 7.13, P<0.001; Figure 2) and zoospores (F(5,44) = 6.12, P<0.001; Figure 3) were counted compared to 0 ppt. The proportion of zoospores that were motile was also found to be significantly lower in salt concentrations greater than 3 ppt compared to the 0 ppt control (F(5,44) = 16.93, P<0.001; Figure 4). However, no effect on zoospore viability was found with no significant difference in the proportion of dead zoospores detected (F(5,44) = 0.89, P = 0.50).

### Infection Experiment

**Figure 5:**
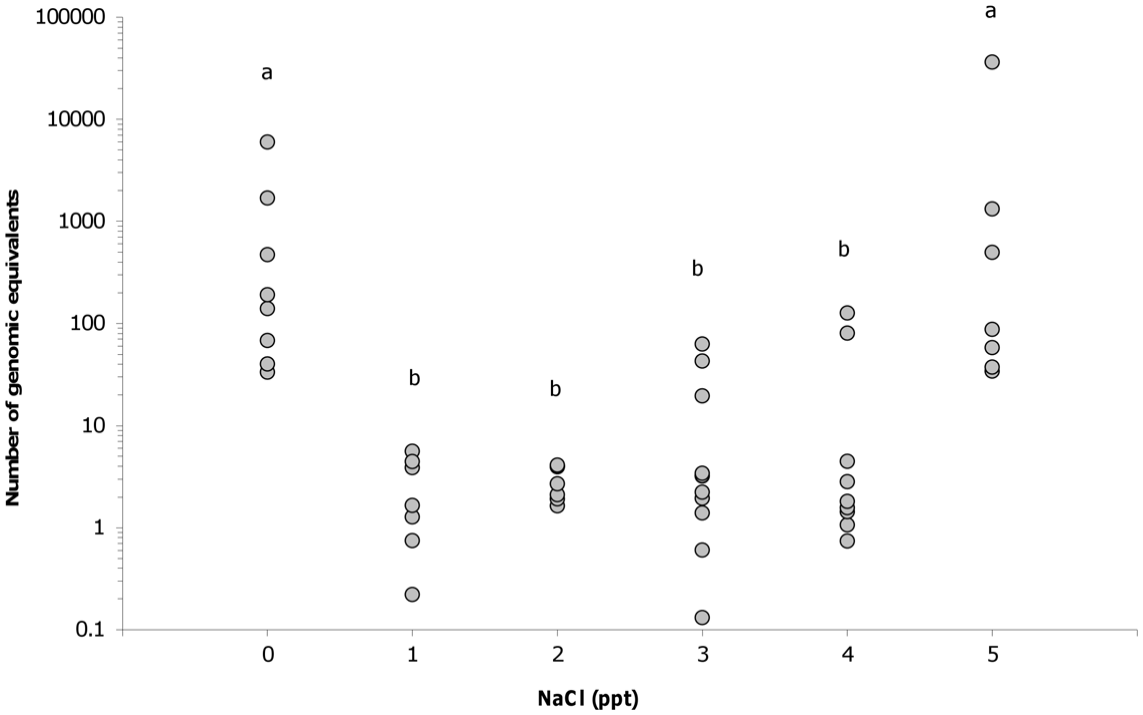
Effect of NaCl on infection load. The number of *Batrachochytrium dendrobatidis* genomic equivalents detected on epidermal swabs from juvenile *Litoria peronii* housed in tanks with water bodies at varying salt concentrations. Different letters indicate significant differences were found between groups.

**Figure 6:**
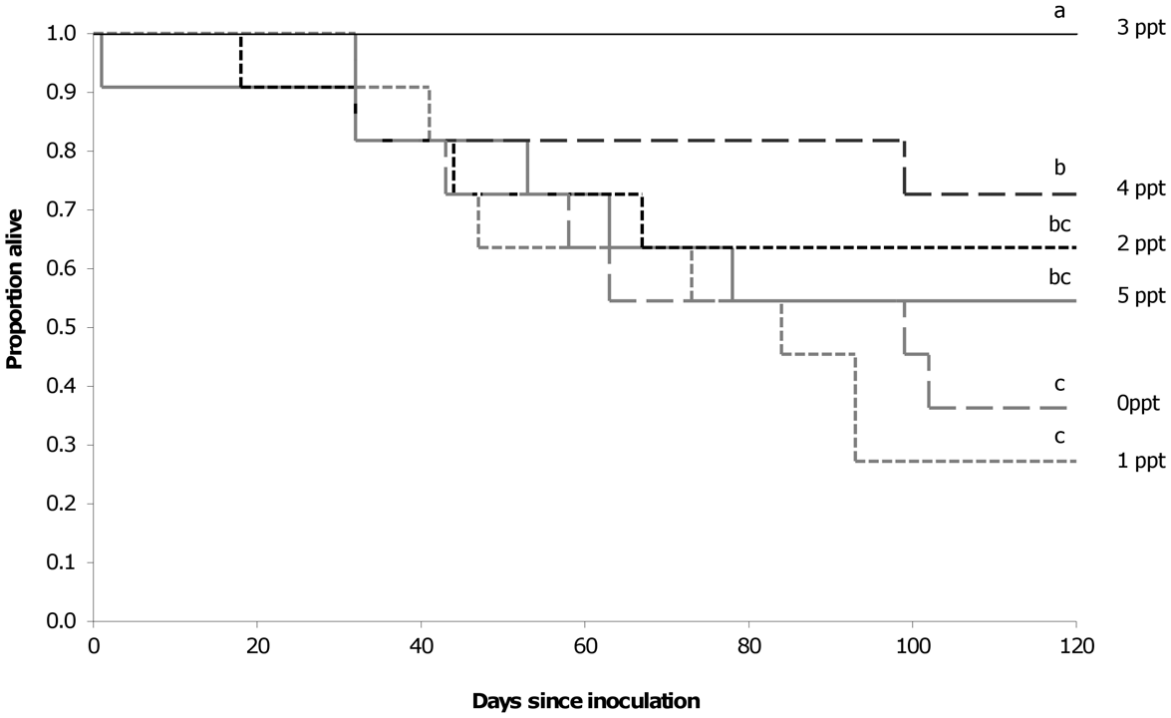
Effect of NaCl on survival rates. Survival curves for juvenile *Litoria peronii* inhabiting tanks with waterbodies of varying salt concentrations (0–5 ppt) following inoculation with *Batrachochytrium dendrobatidis*. Death was assumed following the expression of lethargy and slow righting reflex typical of the terminal stages of chytridiomycosis. Different letters above lines indicate significant differences were found between groups.

All frogs exposed to the chytrid fungus were found to be infected two months after inoculation and all frogs not exposed remained negative for infection. A significant effect of salt was found on the infection loads detected (χ^2^(5) = 34.34, P<0.01), with animals inhabiting 0 and 5 ppt tanks having significantly higher infection loads than the other treatment groups (Figure 5). A significant effect of salt was also found on survival time (χ^2^(5) = 21.53, P = 0.01), with animals in 3 ppt having significantly higher survival rates than all other treatment groups while those in 0 and 1 ppt had the lowest survival rates, significantly more so than those in 3 and 4 ppt (Figure 6). All of the frogs not exposed to the chytrid fungus survived until the end of the experiment, regardless of the salt treatment they were exposed to. Frogs were found to gain up to 0.6 g over the two month period studied, but neither the exposure to salt (F(5,104) = 0.27, P = 0.93) or to chytrid (F(1,104) = 1.0, P = 0.32) was found to have a significant effect on the amount of weight gained.

## Discussion

Exposure to NaCl at concentrations regularly observed in natural lentic frog habitat was not found to affect the survival rate of the chytrid fungus, but did affect growth and infective capacity. Exposure to 4 and 5 ppt in culture media resulted in significantly fewer developing and mature zoosporangia compared to those in 0 ppt, which in turn resulted in the production of significantly fewer zoospores, after 11 days growth. The zoospores exposed to 4 and 5 ppt NaCl were also significantly less motile than those in 0 ppt. These concentrations likely reflect the point at which energy is diverted away from growth and movement, and into osmoregulation. In the current study, exposure of juvenile Peron's tree frogs to 1–4 ppt NaCl also resulted in significantly lower infection loads than those exposed to 0 ppt and significantly higher survival rates in 3 and 4 ppt NaCl. The lower minimum inhibitory concentration of NaCl in the exposure experiment compared to the culture experiment suggests greater negative impacts in host tissue or over a longer time frame. These results show that NaCl does have an inhibitory effect on chytrid growth and dispersal, which in turn determines the outcome of infection.

The growth rate and dispersal ability of a pathogen can directly alter the outcome of infection via effects on infection load dynamics and transmission probability [41]. The rate at which a pathogen grows determines the rate in which infection load within host tissue increases, which in turn determines the time taken to reach disease causing thresholds [42], [43], [44]. The greater the lag time between exposure to the chytrid fungus and the onset of disease, the greater the likelihood of surviving infection through the actions of innate immune responses [16], [19] and seasonally linked temperature changes [45], [46]. In addition, the rate at which the fungus grows can determine the abundance of zoospores in the environment and therefore the likelihood of host exposure and infection [44]. The motility of zoospores will also affect the likelihood of exposure events as actively dispersing keratinophyllic zoospores would have a higher probability of encountering new hosts [47].

As a group, chytrids are known to be less tolerant of salt than other fungal groups due to adaptations to freshwater habitats [28], [48]. Evidence suggests that the amphibian chytrid fungus is an introduced species in Australia [1], [49] and so may not be adapted to thrive in all available habitats where potential hosts occur. Previous work investigating the links between NaCl and this fungus have identified negative correlations between pond salt concentrations and host infection loads, as well as significant inhibitory effects on tadpole infection loads [50]. The results from the current study extend this understanding to include effects on the chytrid life cycle and infection outcomes in post-metamorphic hosts. The finding that NaCl inhibits the negative effects of chytrid infection on susceptible host species provides support for the role of environmental salt manipulations as a potential management tool to mitigate the effects of chytridiomycosis on susceptible host species.

The addition of NaCl to ponds could provide a novel means for preventing further amphibian declines and for allowing reintroduction of susceptible species back into areas where they have disappeared. Although not investigated in the current study, an additional mechanism for the beneficial effects of NaCl on infection outcomes may also be acting. The pathogenesis of chytridiomycosis is linked to reduced absorption of electrolytes due to disruptions to epidermal function, with diseased frogs having considerably lower sodium and chloride plasma concentrations than non-diseased frogs [5], [6]. Although oral electrolyte supplements administered in the terminal stages of chytridiomycosis did not prevent death [6], exposure to environmental NaCl earlier in the infection process may compensate for epidermal dysfunction.

Despite the potential benefits for the mitigation of chytridiomycosis effects through the addition of salt to ponds, there are serious risks associated with the manipulation of environmental salt levels on the host species itself, if it has tolerance limits close to or below the inhibitory concentrations for the chytrid fungus. The results of the current study revealed that NaCl concentrations up to 5 ppt did not affect the growth of Peron's tree frogs over a 2 month period, although effects over a longer time period cannot be ruled out. There were indications that the combination of 5 ppt NaCl and infection had a cumulative effect on infection loads, which may indicate a maximum tolerance limit of salt for this species, resulting in stress effects and/or a direct suppression of immune function [51]. Such negative effects would need to be understood and considered in a management scenario.

Additional considerations would also need to be given to the effect of salt on the rest of the environment. Increasing the salinity of an aquatic environment has the potential to exclude salt sensitive organisms and promote salt tolerant ones, which could substantially affect the structure and function of biological communities [52], [53]. Ultimately, for the use of salt as an antifungal agent in natural frog habitat, the benefits gained by increased survivorship of infected frogs needs to be weighed against the negative effects of salt on both the target and non-target species. Future studies into the potential role of salt as a management tool for chytridiomycosis should be conducted in a more natural setting to allow environmental impacts and interactions to be assessed.
